# Supplementary material for: Effective Biocorrosive Control in Oil Industry Facilities: 16S rRNA Gene Metabarcoding for Monitoring Microbial Communities in Produced Water
Source: Microorganisms. 2023 Mar 27;11(4):846. doi: 10.3390/microorganisms11040846 (PMC10141917; doi:10.3390/microorganisms11040846)
Supplement: Supplementary file 1 [file microorganisms-11-00846-s001.zip › microorganisms-2214874-supplementary.pdf]

Supplementary Materials

Effective Biocorrosive Control in Oil Industry Facilities: 16S rRNA Gene Metabarcoding for Monitoring Microbial Communities in Produced Water

Joyce Dutra [1,2], Glen García [3], Rosimeire Gomes [1], Mariana Cardoso [3], Árley Côrtes [2], Tales Silva [2], Luís de Jesus [2], Luciano Rodrigues [4], Andria Freitas [2], Vinicius Waldow [5], Juliana Laguna [2], Gabriela Campos [2], Monique Américo [2], Rubens Akamine [5], Maíra de Sousa [5], Claudia Groposo [5], Henrique Figueiredo [4], Vasco Azevedo [1,2,3] and Aristóteles Góes-Neto [1,3,*]

[1] Department of Microbiology, Institute of Biological Sciences, Federal University of Minas Gerais, Belo Horizonte 31270-901, MG, Brazil; dutra.engenharia.ambiental@gmail.com (J.D.); rosi.floripes@gmail.com (R.G.); vasco@icb.ufmg.br (V.A.)

[2] Department of Genetics Ecology and Evolution, Institute of Biological Sciences, Federal University of Minas Gerais, Belo Horizonte 31270-901, MG, Brazil; arleyrezcort@gmail.com (Á.C.); talesfdasilva@gmail.com (T.S.); luiislimma@gmail.com (L.d.J.); andria.sfreitas@gmail.com (A.F.); jujulaguna@gmail.com (J.L.); gabrielamunis24@gmail.com (G.C.); moniquefamerico@gmail.com (M.A.)

[3] Departments of Bioinformatic, Institute of Biological Sciences, Federal University of Minas Gerais, Belo Horizonte 31270-901, MG, Brazil; glen.yupanqui@gmail.com (G.G.); marianascardoso@yahoo.com.br (M.C.)

[4] Department of Veterinary Medicine, Faculty of Veterinary, Federal University of Minas Gerais, Belo Horizonte 31270-901, MG, Brazil; lsantosrodrigues@gmail.com (L.R.); figueiredoh@icb.ufmg.br (H.F.)

[5] Petrobras Research and Development Center (CENPES), Petrobras, Rio de Janeiro 21941-915, RJ, Brazil; vinicius.waldow@petrobras.com.br (V.W.); akamine@petrobras.com.br (R.A.); mpsousa@petrobras.com.br (M.d.S.); claudiagroposo@gmail.com (C.G.)

* Correspondence: arigoesneto@icb.ufmg.br

Table S1. Quantification of Acid-Producing Bacteria (APB), Sulfate-Reducing Bacteria (SRB), and General Anaerobic Heterotrophic Bacteria (GANB) in Produced Water (PW) samples.

**Microbial Group**	**Quantification (NMP/mL)**	
	**p_1.00**	**p_2.75**
APB	4.3×10^6	4.3×10^6
SRB	2.3×10^5	2.3×10^5
GANB	2.3×10^5	2.3×10^5

Sample Points (height): 1.00 m (p_1.00); 2.75 m (p_2.75).

For quantification, mathematical equations were used, based on the Poisson's law. It is assumed that bacteria are distributed in a dilution following a Poisson distribution. Thus, the average number of bacteria can be calculated by equation 1.

$$P_0 = e^{-\lambda} \qquad (Eq. 1)$$

Therefore, applying the equation above for more than one dilution, equation 2 is developed:

$$\sum_{j=1}^{K} \frac{g_j m_j}{1 - exp(-\lambda m_j)} = \sum_{j=1}^{K} t_j m_j \qquad (Eq. 2)$$

Where:

λ = average number of bacteria (MPN);
P_0 = rate of negative bottles relative to the total number of bottles;
K-j = number of dilutions;
g-j = number of positive tubes in the j-th dilution;
m-j = volume of sample applied to each tube in the j-th dilution;
t-j = number of tubes in the j-th dilution

Table S2. Relative abundance of phylum by sample type

Phylum	APB_1.00m	APB_2.75m	SRB_1.00m	SRB_2.75m	GANB_1.00m	GANB_2.75m	PW_1.00m	PW_2.75m
Acidobacteriota	0	0	0	0	0	0	0.0210	0.0114
Actinobacteriota	0	0.0002	0.0003	0	0	0	0.1632	0.2375
Aenigmarchaeota	0	0	0	0	0	0	0.0022	0.0019
Bacteroidota	0.0024	0.1078	10.4793	4.7494	0.0025	0.0044	1.8795	1.5274
Bdellovibrionota	0	0	0	0	0	0	0.0007	0.0019
Caldatribacteriota	0	0	0.0004	0.0005	0	0	0.7346	0.6207
Caldisericota	0	0	0.0001	0	0	0	0.2063	0.1612
Calditrichota	0	0	0.0077	0.0027	0	0	0.0016	0.0023
Campylobacterota	9.1107	1.8034	0.5259	21.1318	36.7056	20.6202	1.5413	1.5838
Chloroflexi	0	0.0003	0.0059	0.0045	0	0	2.5047	2.2710
Cloacimonadota	0.0005	0.0003	0.5423	0.1963	0.0004	0.0004	19.3182	16.5178
Cyanobacteria	0	0	0	0	0	0	0.0433	0.0140
Deferribacterota	0	0	0	0	0	0	0.0013	0.0007
Desulfobacterota	14.9059	28.5632	6.5649	35.2637	16.8342	30.6903	10.2219	9.4864
Elusimicrobiota	0	0	0	0	0	0	0.0022	0.0040
Euryarchaeota	0	0	0.0001	0	0	0	0.1324	0.1778
Fermentibacterota	0	0	0	0	0	0	0.0426	0.0586
Firmicutes	9.4256	8.5137	26.1685	12.0253	0.0174	0.0321	46.5111	51.5058
Halanaerobiaeota	8.6618	20.2162	0.0064	0.0043	0.0011	0.0022	2.7194	2.6634
Halobacterota	0	0.0005	0.0110	0.0059	0.0004	0.0002	7.7203	6.6823
Marinimicrobia (SAR406 clade)	0	0	0.1539	0.0200	0	0	0.9824	0.7457
Nanoarchaeota	0	0	0.0001	0	0	0	0.0431	0.0560
Patescibacteria	0	0	0.0004	0.0160	0	0.0002	0.4346	0.5411
Proteobacteria	0.3043	0.0874	44.2304	16.3818	0.0587	0.5543	0.4654	0.4382
Spirochaetota	15.0545	0.0291	0.0522	0.0278	0	0.0002	0.1384	0.1134
Sumerlaeota	0	0	0	0	0	0	0.0036	0.0040
Synergistota	42.5196	40.6769	3.7111	4.0607	46.2892	48.0675	2.5353	2.8335
Thermoplasmatota	0	0	0	0	0	0	0.0004	0.0007
Thermotogota	0.0076	0.0006	7.1979	5.7181	0	0	1.2853	1.4214
Unclassified	0.0070	0.0005	0.3409	0.3912	0.0905	0.0279	0.3085	0.2987

Verrucomicrobiota	0	0	0	0	0	0	0.0353	0.0175

Table S3. Relative abundance of genus by sample type

Genus	APB_1.00m	APB_2.75m	SRB_1.00m	SRB_2.75m	GANB_1.00m	GANB_2.75m	PW_1.00m	PW_2.75m
Acetobacterium	0	0.0002	0.0066	0.0035	0	0	2.7297	3.3221
Acetomicrobium	0	0	0	0	0	0	0.0009	0.0014
Acinetobacter	0.0049	0	0.0004	0	0.0005	0	0	0.0005
ADurb.Bin120	0	0.0003	0.0017	0.0013	0	0	1.6647	1.5080
Alkalibacter	0	0	0.0003	0	0	0	0.0183	0.0117
Amnipila	0	0	0	0	0	0	0.0036	0.0049
Anaerofustis	0	0	0.0009	0	0	0	0.0196	0.0224
Anaerostignum	0	0	0	0	0	0	0.0011	0.0026
Anaerovorax	0	0	0.0239	0.0168	0	0	0.2877	0.1960
Bacillus	0.0011	0.0014	0.0016	0.0003	0.0016	0.0038	0.0004	0.0047
bacterium YC-ZSS-LKJ23 (no Genus in SILVA)	0	0	0	0	0	0	0.0083	0.0093
Bacteroides	0	0	0	0	0	0	0.0069	0.0117
Brevundimonas	0.0005	0	0.0001	0	0.0011	0	0	0
Caldisericum	0	0	0	0	0	0	0.0013	0.0023
Caminicella	0	0	0	0	0	0	0.0462	0.0217
Candidatus Diapherotrites archaeon ADurb.Bin253	0	0	0	0	0	0	0.0013	0.0033
Candidatus Marispirochaeta	0	0	0.0123	0.0032	0	0	0.0025	0.0016
Candidatus Riegeria	0	0	0.0004	0.0005	0	0	0.0007	0.0021
Caproiciproducens	0	0	0	0	0	0	0.0007	0.0023
Castellaniella	0.0011	0.0025	0.0009	0.0024	0.0009	0.0024	0.0025	0.0040
Christensenellaceae R-7 group	0	0	0.0054	0.0075	0	0	0.0210	0.0247
Cloacibacterium	0.0019	0.0051	0.0014	0.0069	0.0024	0.0033	0.0045	0.0033
Clostridium	0.0011	0.0002	0.0433	0	0	0.0002	0.0283	0.0495
Curvibacter	0.0325	0.0014	0.0003	0.0008	0	0	0.0259	0.0054
Cyanobium PCC-6307	0	0	0	0	0	0	0.0013	0.0021
Defluviitaleaceae UCG-011	0	0	0.0607	0	0	0	0.0013	0.0002
Dehalobacterium	0	0	0	0	0	0	0.0125	0.0119
Desulfatiglans	0	0	0	0.0005	0	0	0.0234	0.0224

Desulfobacter	0.0005	0.1552	2.6049	1.5572	1.3746	0.0992	0.1049	0.0523
Desulfobacterium	0	0	0.0027	0.0013	0	0.0004	0.0004	0.0005
Desulfobulbus	0	0	0.0039	0.0077	0.0033	0.0002	0.0156	0.0236
Desulfocella	0	0	0	0	0	0	0.0004	0.0007
Desulfocurvus	0	0	0.0063	0.0203	0	0	0.0087	0.0072
Desulfomicrobium	0	0	0.0316	0.0166	0	0	0.0250	0.0112
Desulfoplanes	0	0	0.2220	0.0863	0	0	0.5549	0.3285
Desulfotignum	0	0	0	0	0	0	0.0069	0.0019
Desulfovibrio	14.8859	28.3678	1.9808	32.8451	15.3855	30.5361	3.6717	6.0362
Desulfuromonas	0	0	0	0	0	0	0.0060	0.0026
Dethiosulfatarculus	0	0	0.0086	0.0037	0	0	0.0225	0.0147
Dethiosulfatibacter	0.2144	0.0022	3.1225	1.4224	0.0004	0.0018	25.4941	23.9479
Dethiosulfovibrio	42.4605	40.6651	3.2809	3.8513	46.2540	48.0449	0.1083	0.1969
DMER64	0	0	0.0001	0	0	0	0.0306	0.0229
EBM-39	0	0	0.1762	0.1047	0	0	0.0098	0.0086
Endomicrobium	0	0	0	0	0	0	0.0022	0.0040
Enterococcus	0	0.0025	0	0	0	0.0004	0	0
Ercella	0	0	0.0036	0.0013	0	0	0.0058	0.0084
Escherichia-Shigella	0.0815	0.0332	0.0099	0.0176	0.0460	0.0359	0.0158	0.0376
Eubacterium	0	0	0	0	0	0	0.0029	0.0051
Fastidiosipila	0	0	0	0	0	0	0.0011	0.0061
Flexistipes	0	0	0	0	0	0	0.0013	0.0007
Fusibacter	9.0953	8.4322	0.1182	0.5544	0.0004	0.0027	5.8561	11.9940
Geotoga	0.0076	0.0003	0.2649	2.5259	0	0	0.1158	0.1440
Guggenheimella	0	0	0.0901	0.0259	0.0002	0	0.4815	0.8740
Haemophilus	0	0	0	0	0	0	0	0.0035
Halanaerobium	8.6618	20.2162	0.0064	0.0043	0.0011	0.0022	2.7141	2.6560
Halarcobacter	0.1505	0.0846	0.3652	15.4963	25.3623	20.0798	0.0288	0.0317
Halodesulfovibrio	0	0.0382	1.6329	0.6677	0	0.0007	0.0004	0.0021
Halomonas	0.0796	0	0.1225	0.0601	0	0	0.0172	0.0100
Irregularibacter	0	0	0.0172	0.0088	0	0	0	0
JGI 0000069-P22 (no Genus in SILVA)	0	0	0	0	0	0	0.0025	0.0012
JTB215	0.0022	0.0011	15.0371	8.9428	0.0004	0.0004	1.4987	1.6438

Lactiplantibacillus	0.0097	0.0006	0	0	0	0	0	0.0168
Lactococcus	0	0.0083	0.0001	0.0013	0.0004	0.0002	0	0
Lentimicrobium	0	0	0	0	0	0	0.0047	0.0019
Levilinea	0	0	0	0	0	0	0.0080	0.0072
LNR A2-18	0.0005	0.0002	0.0124	0.0072	0.0004	0.0002	14.2733	11.9329
Malaciobacter	0	0	0	0	0	0	0.0196	0.0103
Marinilabilia	0	0	0.1154	0.0534	0	0	0.0007	0
Marinobacter	0	0.0005	0.0752	0.0481	0.0007	0.0053	0.0087	0.0159
Marinobacterium	0	0.0006	43.8161	16.0491	0.0013	0.0711	0.0337	0.0273
Marispirillum	0	0	0	0	0	0.1400	0.0002	0.0002
Melioribacter	0	0	0	0	0	0	0.0154	0.0105
Mesotoga	0	0	0.0009	0.0003	0	0	0.0975	0.1027
Methanobacterium	0	0	0	0	0	0	0.0424	0.0691
Methanobrevibacter	0	0	0	0	0	0	0.0076	0.0203
Methanocalculus	0	0.0003	0.0004	0	0	0.0002	0.6685	0.6424
Methanococcus	0	0	0	0	0	0	0.0362	0.0497
Methanoculleus	0	0	0	0	0	0	0.0013	0.0016
Methanofollis	0	0	0	0	0	0	0.0074	0.0159
Methanohalophilus	0	0	0	0	0	0	0.0007	0.0019
Methanolacinia	0	0	0	0	0	0	0.0208	0.0299
Methanolinea	0	0	0	0	0	0	0.0344	0.0201
Methanolobus	0	0	0.0044	0.0011	0	0	0.0063	0.0070
Methanomicrobium	0	0	0	0	0	0	0.0013	0.0037
Methanoplanus	0	0	0.0013	0	0.0004	0	0.4636	0.3932
Methanothrix	0	0	0.0037	0.0043	0	0	6.2398	5.1685
Methanosarcina	0	0	0.0007	0.0003	0	0	0.0069	0.0063
Methanothermobacter	0	0	0	0	0	0	0.0065	0.0082
Neochlamydia	0	0	0	0	0	0	0.0042	0.0044
NK4A214 group	0	0	0.0001	0	0	0	0.1239	0.3101
Oceanotoga	0	0.0002	6.8314	3.1767	0	0	0.0029	0.0021
Oenococcus	0	0.0003	0	0	0	0	0	0
Ornatilinea	0	0	0.0024	0.0016	0	0	0.0042	0.0075
Oscillibacter	0	0	0	0	0	0	0.0092	0.0035
Paenibacillus	0.0027	0.0050	0.0020	0.0109	0.0049	0.0095	0.0121	0.0063

Parabacteroides	0	0	0.0010	0.0056	0	0	0.0009	0
Paracoccus	0.0049	0.0044	0.0017	0.0099	0.0027	0.0120	0.0054	0.0096
Paramaledivibacter	0	0	0	0	0	0	0.0016	0.0007
Pelolinea	0	0	0.0007	0.0011	0	0	0.2529	0.2385
Pelospora	0	0	0	0	0	0	0.0054	0.0030
PeM15	0	0	0	0	0	0	0.0060	0.0033
Peptoclostridium	0	0	1.1354	0.1803	0	0	0.0067	0.0028
Prolixibacter	0	0.0022	0.0023	0	0	0	0.0158	0.0138
Proteiniphilum	0	0.0581	0.0073	0.3346	0	0.0002	0.0636	0.0656
Pseudomonas	0	0	0	0	0	0	0.0007	0.0007
Ralstonia	0.0953	0.0414	0.0240	0.0443	0.0016	0.0031	0.3281	0.2777
Roseimarinus	0	0	0.0144	0.0013	0	0	0.0150	0.0098
SC103	0	0.0002	0.0094	0.0016	0	0	0.8489	0.9032
SCGC AAA011-D5 (no Genus in SILVA)	0	0	0	0	0	0	0.0167	0.0168
SCGC AAA286-E23 (no Genus in SILVA)	0	0	0.0001	0	0	0	0.0040	0.0233
Sediminispirochaeta	0	0	0.0183	0.0083	0	0	0.0054	0.0030
Sphaerochaeta	15.0545	0.0291	0.0172	0.0144	0	0.0002	0.0748	0.0721
Staphylococcus	0.0027	0.0030	0.0004	0	0.0058	0.0004	0.0004	0.0028
Streptococcus	0.0089	0.0070	0.0034	0.0147	0.0031	0.0124	0.0058	0.0114
Sulfurimonas	0	0	0	0	0	0	0.0060	0.0042
Sulfurospirillum	7.8761	1.2878	0.0017	0.0013	0	0	0.4152	0.2784
Sunxiuqinia	0	0	0.0649	0.3341	0	0.0002	0	0.0005
Syntrophomonas	0	0	0	0	0	0	0.1100	0.0775
Syntrophotalea	0.0005	0.0014	0.0250	0.0187	0.0649	0.0492	5.1139	2.5008
Tangfeifania	0	0	0.0030	0.0005	0	0	0.1000	0.1356
Tepidiphilus	0	0	0	0	0	0	0.0016	0.0012
Thauera	0	0	0	0	0	0	0.0018	0.0005
Thermomonas	0	0.0005	0	0	0.0002	0	0.0009	0.0028
Thermovirga	0	0.0002	0.0316	0.0144	0	0	2.0049	1.9268
Unclassified	1.2611	0.5394	18.5022	11.3950	11.4792	0.8810	22.7037	21.1829
Vulcanibacillus	0	0	0	0	0	0	0.0031	0.0042

Statistical analyzes were performed with the R packages: *stats* v4.2.1 and *car* v3.1.1. The plots of the box-plots were made with the *ggplot2* v3.4.0 package.

The Shannon (H), Dominance (D) and Equitability (J) alpha-diversity indices of the replicates of the APB, SRB, GANB and PW samples (Table 1) follow a normal distribution (Shapiro-Wilk) with the exception of the Dominance indices of the APB sample (Table 2). However, since it was very close to alpha (α = 0.05), it was decided to analyze the possible differences in the indices using the ANOVA test. Additionally, the indices of Table 1 are graphically presented in box-plots (Figures S1, S2 and S3).

Table S4. Values of the Shannon (H), Dominance (D) and Equitability (J) diversity indices of the replicates of the APB, SRB, GANB and PW samples.

Sample	Dominance (D)	Shannon (H)	Equitability (J)
APB-1-1	0.49	1.018	0.3292
APB-1-2	0.289	1.366	0.4639
APB-1-3	0.2599	1.606	0.482
APB-2.75-1	0.5395	0.7257	0.2511
APB-2.75-2	0.5047	0.7652	0.2272
APB-2.75-3	0.4873	0.7996	0.2516
SRB-1-1	0.2621	1.79	0.4338
SRB-1-2	0.3322	1.563	0.3759
SRB-1-3	0.4627	1.353	0.3332
SRB-2.75-1	0.355	1.307	0.3966
SRB-2.75-2	0.214	1.887	0.4731
SRB-2.75-3	0.1986	1.868	0.4582
GANB-1-1	0.3473	1.158	0.3934
GANB-1-2	0.3363	1.186	0.3958
GANB-1-3	0.6799	0.6152	0.2272
GANB-2.75-1	0.4717	0.8405	0.2908
GANB-2.75-2	0.462	0.8804	0.2939
GANB-2.75-3	0.3622	1.066	0.3399
PW-1-1	0.1644	2.384	0.5248
PW-1-2	0.162	2.34	0.505
PW-1-3	0.1741	2.279	0.4887
PW-2.75-1	0.1156	2.57	0.5522
PW-2.75-2	0.1862	2.144	0.4687
PW-2.75-3	0.1969	2.127	0.4788

Table S5. Normality test of alpha diversity indices by type of sample.

Sample Type	Shannon (H) *p-value*	Dominance (D) *p-value*	Equitability (J) *p-value*
APB	0.1984	0.04769	0.1101
SRB	0.209	0.6428	0.8228
GANB	0.5532	0.1077	0.5334
PW	0.6644	0.3796	0.7506

All the ANOVA tests suggested that there are statistically significant differences between the types of samples (Table 3), as well as the Levene tests indicated that these data groups have similar variances.

Table S6. ANOVA and Levene test of alpha-diversity indices by type of sample.

	ANOVA *p-value*	Levene *p-value*
Shannon (H)	4.70E-08	0.3265
Dominance (D)	4.34E-04	0.2958
Equitability (J)	1.10E-03	0.07618

The Tukey test was performed to observe these significant differences by pairs (Table 4), where it

was found that the Shannon indices of the PW samples against the other types of samples are different (PW-APB: p = 0.0000003, PW-SRB: p = 0.0011662 and PW-GANB: p = 0.0000001), there were also significant differences between PW and the APB and GANB samples for the Dominance indices (PW-APB: p = 0.0014067 and PW-GANB: p = 0.0007951) and Equitability (PW-APB: p = 0.0032440 and PW-GANB: p = 0.0018114).

Table S7. Tukey Post-Hoc Test.

**Pairs**	**Shannon (H)** *p-adj*		**Dominance (D)** *p-adj*		**Equitability (J)** *p-adj*	
PW-APB	0.0000003	(*)	0.0014067	(*)	0.0032440	(*)
GANB-APB	0.9340887		0.9943321		0.9939472	
SRB-APB	0.0051133	(*)	0.1886823		0.2758206	
GANB-PW	0.0000001	(*)	0.0007951	(*)	0.0018114	(*)
SRB-PW	0.0011662	(*)	0.1271232		0.1610804	
SRB-GANB	0.0013365	(*)	0.1211039		0.1819608	

(*) There is significant difference

Below are presented the data in Box-Plot

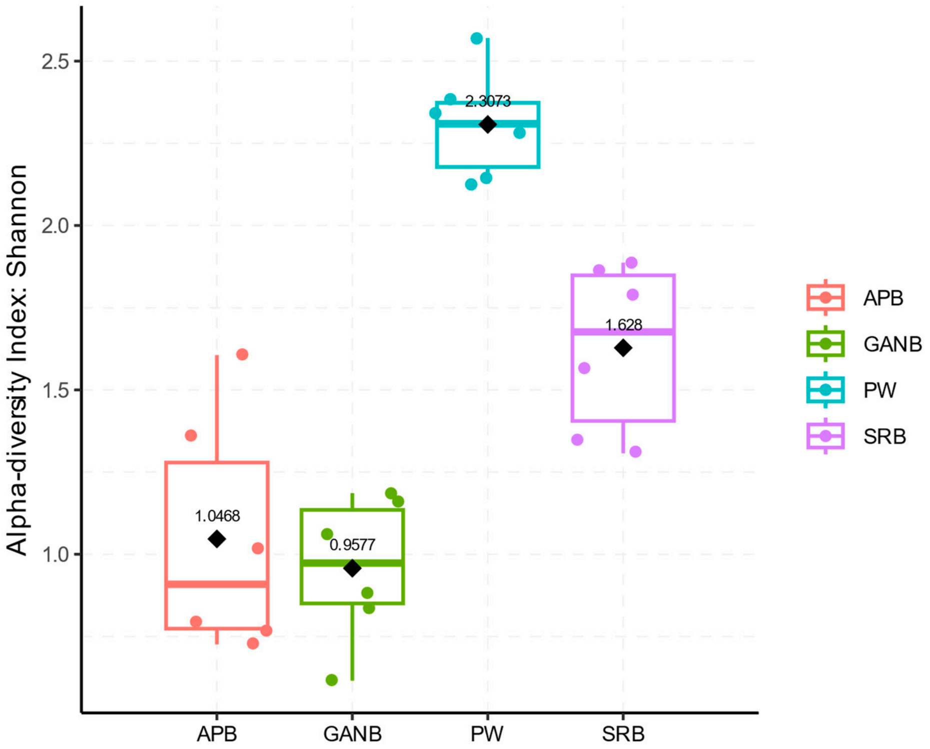

Figure S1. Box plot of the Shannon indices for the APB, SRB, GANB and PW samples

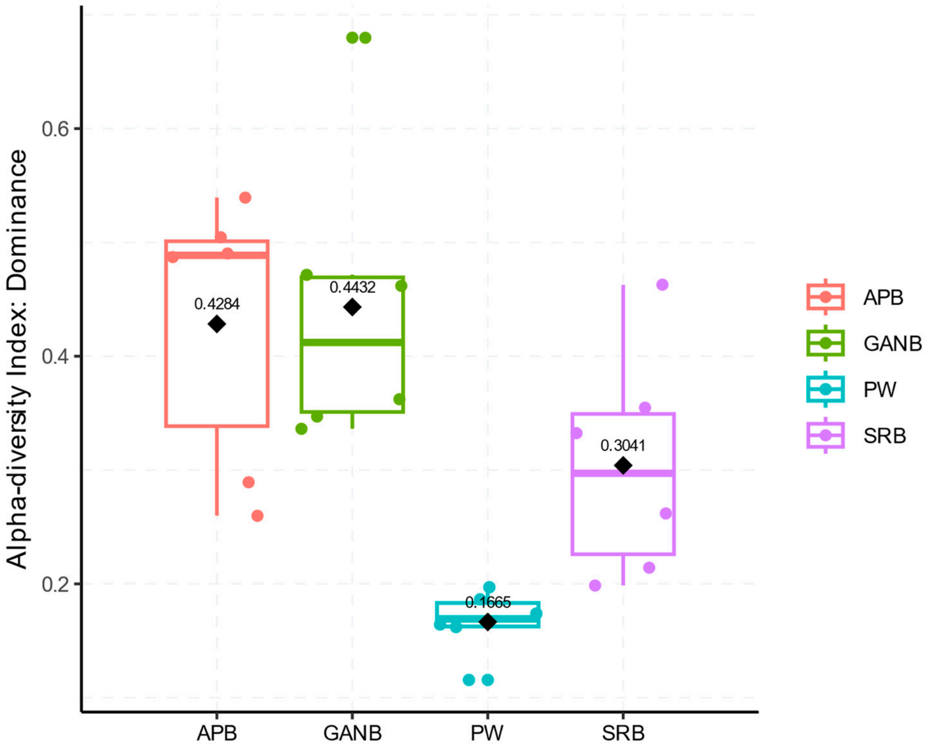

Figure S2. Box plot of the Dominance indices for the APB, SRB, GANB and PW samples

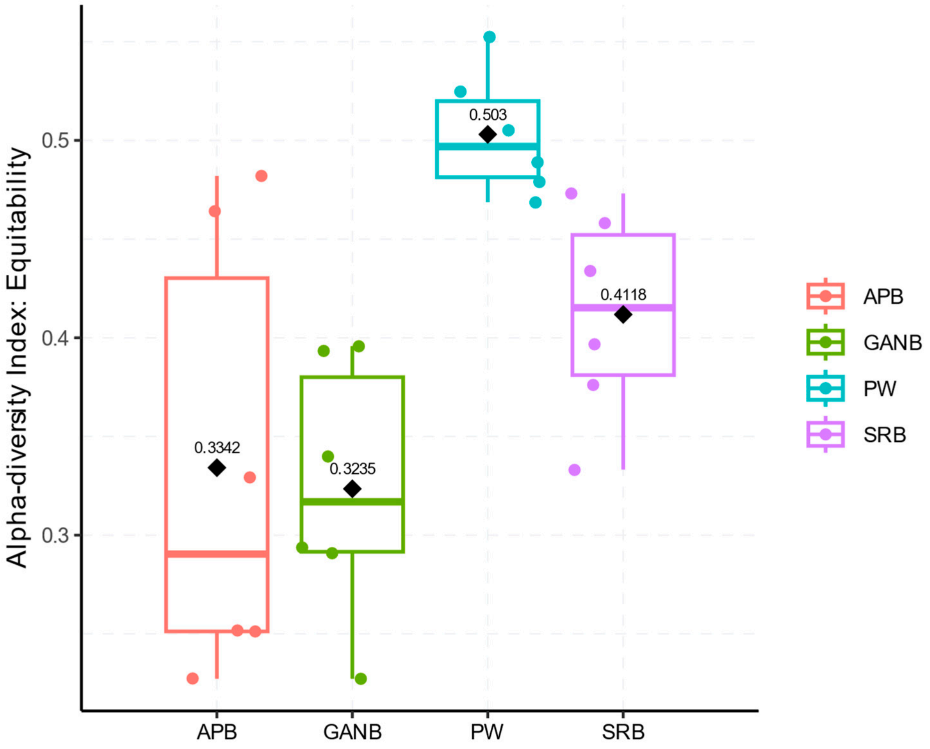

Figure S3. Box plot of the Equitability indices for the APB, SRB, GANB and PW samples
